# Diurnal rhythms in varicella vaccine effectiveness

**DOI:** 10.1172/jci.insight.184452

**Published:** 2024-09-03

**Authors:** Dana Danino, Yoav Kalron, Jeffrey A. Haspel, Guy Hazan

**Affiliations:** 1 Faculty of Health Sciences, Ben Gurion University, Beer Sheva, Israel.; 2 Pediatric Infectious Diseases Unit, Saban Children Hospital, Soroka University Medical Center, Beer Sheva, Israel.; 3 Division of Pulmonary and Critical Care Medicine, Department of Internal Medicine, Washington University School of Medicine, St. Louis, Missouri, USA.; 4 Pediatric Pulmonary Unit, Saban Children Hospital, Soroka University Medical Center, Beer Sheva, Israel.

**Keywords:** Vaccines, Bacterial vaccines

## Abstract

**BACKGROUND:**

Immune processes are influenced by circadian rhythms. We evaluate the association between varicella vaccine administration time of day and vaccine effectiveness.

**METHODS:**

A national cohort, children younger than 6 years, were enrolled between January 2002 and December 2023. We compared children vaccinated during morning (7:00–10:59), late morning to afternoon (11:00–15:59), or evening hours (16:00–19:59). A Cox proportional hazards regression model was used to adjust for ethnicity, sex, and comorbidities. The first varicella infection occurring at least 14 days after vaccination and a second dose administration were treated as terminal events.

**RESULTS:**

Of 251,141 vaccinated children, 4,501 (1.8%) experienced breakthrough infections. Infection rates differed based on vaccination time, with the lowest rates associated with late morning to afternoon (11:00–15:59), HR 0.88, 95% CI 0.82–0.95, *P* < 0.001, and the highest rates with evening vaccination (16:00–19:59), HR 1.41, 95% CI 1.32–1.52, *P* < 0.001. Vaccination timing remained significant after adjustment for ethnicity, sex, and comorbidities. The association between immunization time and infection risk followed a sinusoidal pattern, consistent with a diurnal rhythm in vaccine effectiveness.

**CONCLUSION:**

We report a significant association between the time of varicella vaccination and its clinical effectiveness. Similar association was observed with the COVID-19 vaccine, providing proof of concept consistent with a diurnal rhythm in vaccine effectiveness.

## Abstract 2

Varicella vaccine effectiveness in preventing breakthrough infections among children varies by vaccination time of day, with evening vaccinations showing the lowest effectiveness.

## Introduction

Circadian rhythms are 24-hour oscillations in biological function emanating from a genetically encoded molecular clock that regulates gene expression and thereby organizes cellular functions into daily cycles (1, 2). Basic studies, examining clock regulation of immune processes that contribute to vaccination, described diurnal variations in immune responses (3, 4). These include dendritic cells’ activation at the site of intramuscular injection (5), trafficking of dendritic cells and lymphocytes to the lymph node (6), selection of antigen-specific T and B cells, and effector functions (7, 8). Studies suggested a correlation between the timing of vaccine administration during the day and vaccine effectiveness (9, 10). However, these studies were constrained by their limited sample sizes and the use of serologic titers as the outcome measure, which restricted the generalization for clinical effectiveness. A recent study examining the correlation between vaccination timing and the efficacy of COVID-19 vaccine doses suggested a sinusoidal pattern in infection risk, aligning with diurnal biological rhythms. This study, conducted amid a mass vaccination campaign in a pandemic setting, revealed that the lowest rates of breakthrough infections were associated with vaccinations administered from late morning to early afternoon, while the highest rates occurred with evening vaccinations (11). We aimed to evaluate the association between vaccination time of day and vaccine effectiveness beyond the COVID-19 vaccine utilizing big data from a nationwide childhood varicella vaccination program. Therefore, we analyzed the time of vaccination in association with varicella breakthrough infection.

## Results

**Figure 1:**
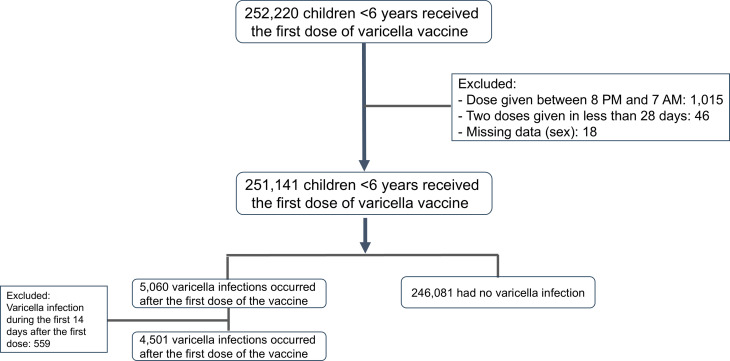
Study population.

**Figure 2:**
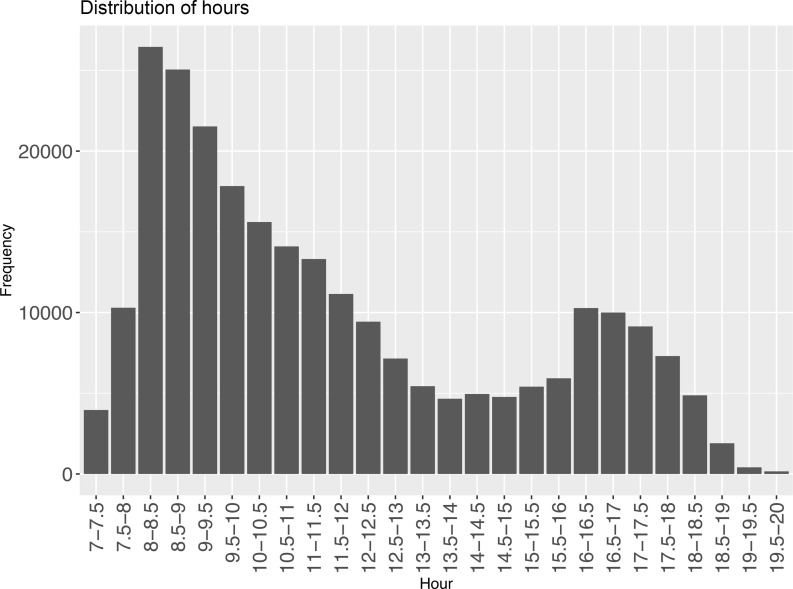
Varicella vaccine timing across the day.

A total of 251,141 children under the age of 6 years old (Figure 1) had timestamps recorded for the first dose of varicella immunization. The distribution of patient immunization times is presented in Figure 2. Based on this distribution, we compared varicella breakthrough infections in patients receiving their first dose of the vaccine in the morning (7:00–10:59, *n* = 134,841), afternoon (11:00–15:59, *n* = 72,223), or evening (16:00–19:59, *n* = 44,077). As a group, vaccinated children had a mean age of 1.2 years (SD 0.6), 51.23% were male, the majority were Jewish (94.4%), 0.56% were defined as immunodeficient, and 8.41% had either obesity diagnosis or weight above the 95th percentile for age and sex (Table 1).

The group of children immunized in the evening exhibited advanced chronological age and higher rates of Jewish ethnicity and obesity relative to both their morning-vaccinated counterparts and those immunized during the late morning to early afternoon. In addition, the late morning to early afternoon vaccination group demonstrated a higher proportion of female children and non-Jewish ethnicity compared with the morning-vaccinated cohort. Notably, a higher proportion of immunodeficiency diagnoses was observed in children immunized during the morning compared with children immunized in the evening (Table 1).

**Figure 3:**
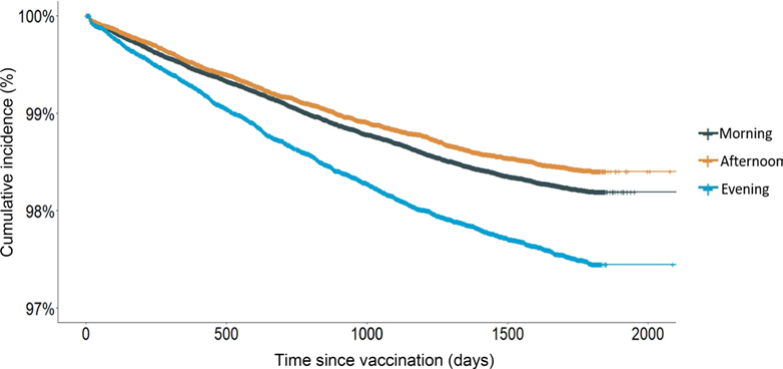
Varicella infection-free survival in children by vaccination time of day.

Overall, children had a varicella breakthrough infection rate of 1.8% (4,501/251,141) after 1 dose of varicella immunization, similar to the 2.1% and 1.56% varicella breakthrough infection rates found in a nationwide retrospective investigation in Taiwan and Beijing, respectively (12, 13). The number of varicella infections per week of the year was similar across the 3 groups of vaccine administration times (Supplemental Figure 1; supplemental material available online with this article; https://doi.org/10.1172/jci.insight.184452DS1). Children immunized in the late morning to early afternoon had less frequent breakthrough infections than children vaccinated in the morning (HR 0.88, 95% CI 0.82–0.95, *P* < 0.001), while those vaccinated in the evening had higher rates of breakthrough infection (HR 1.41, 95% CI 1.32–1.52, *P* < 0.001) (Table 2 and Figure 3).

**Figure 4:**
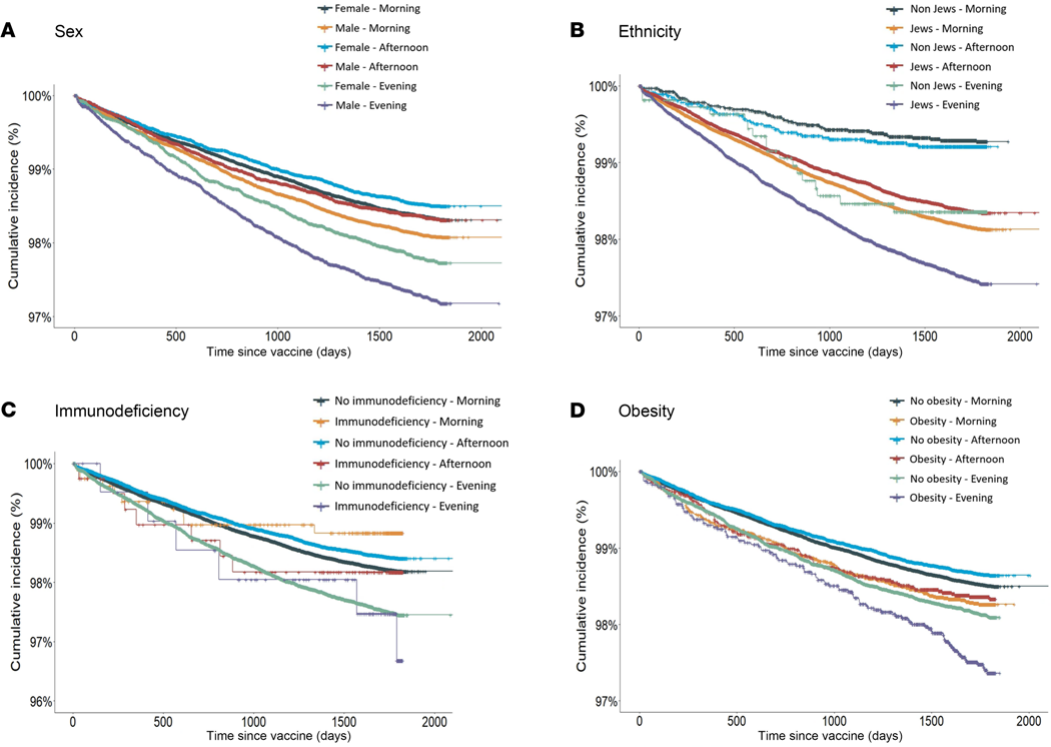
Varicella infection-free survival in children by vaccination time of day. Stratified by sex (**A**), ethnicity (**B**), immunodeficiency (**C**), obesity (**D**).

Obesity, Jewish ethnicity, and male sex were all associated with lower vaccine effectiveness (Supplemental Table 2). However, the difference in effectiveness by vaccination time of day remained significant after stratification by sex, ethnicity, immunodeficiency status, and obesity (Figure 4, A–D).

**Figure 5:**
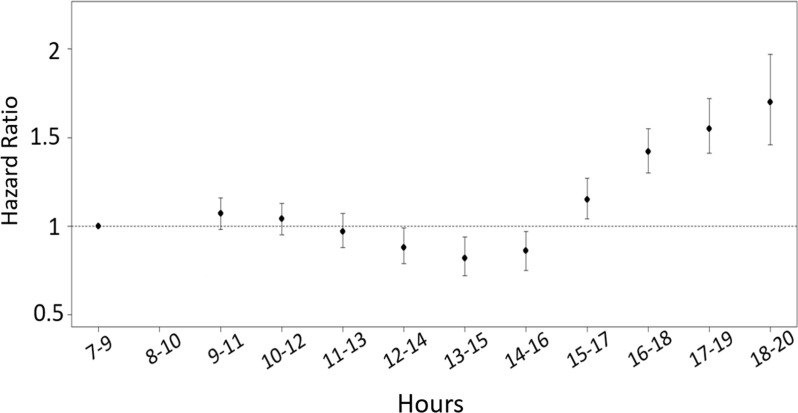
Diurnal rhythms in varicella vaccine effectiveness.

Using Cox multivariate regression to adjust for ethnicity, sex, immunodeficiency, and obesity, we examined how the impact of vaccination time changes throughout the day. Relative to immunizations performed between 8:00 and 9:59, the HR for breakthrough infections oscillated in a sinusoidal pattern, with the lowest risk afforded by late morning to early afternoon vaccination times and the highest risk associated with evening vaccination times (Figure 5).

The sinusoidal contour of this effect suggests a diurnal rhythm in varicella vaccine efficacy based on time of administration.

## Discussion

Our data reveal a notable association between the timing of varicella vaccination and its clinical efficacy regarding varicella breakthrough infection. The correlation between vaccination timing and infection risk exhibited a sinusoidal pattern, indicating a diurnal biological rhythm in vaccine efficacy. Specifically, children immunized during the late morning to early afternoon experienced fewer breakthrough infections compared with those vaccinated in the evening.

Circadian regulation seems to influence various stages of the vaccination process (14). Basic science investigations have documented diurnal variations in immune responses to antigenic challenges. While many human studies examining the effect of vaccine administration time have primarily focused on immunological responses, particularly antibody titers, as the primary endpoint, the findings have been inconsistent. Some reports have suggested morning vaccination as optimal, while others have found no significant difference between morning and afternoon vaccination (9, 15–18). However, it is worth noting that antibody titers have been shown to be a poor surrogate for vaccine efficacy (19, 20). The first study that examined the association between vaccine effectiveness and the time of day when vaccination occurred was conducted during the unique circumstances of a global health crisis, the COVID-19 pandemic vaccination response, leveraging population-based data. This study detected a diurnal rhythm of COVID-19 vaccine efficacy (11). We managed to expand this association in a different clinical setting of a childhood immunization program and with a different vaccine-preventable pathogen, showing similar optimal vaccine efficacy of late morning to early afternoon vaccination, associated with fewer breakthrough infections.

Human populations have less aligned circadian rhythms than other organisms, making it challenging to observe how these rhythms affect medical interventions in real-world settings (21, 22). Our study population of children under the age of 6 years old enabled us to minimize the possibility of a diverse lifestyle with routine exposure to light at night that might alter the phase of the circadian clock.

High vaccine coverage is required to ensure population protection; however, recent studies have found an increase in parents’ vaccine hesitancy and a decline in routine childhood immunization rates, negatively influenced by social media (23, 24). One approach to overcome this phenomenon and motivate parents to vaccinate their children is by increasing vaccine accessibility through extended vaccine clinic operating hours after working hours. Although increasing clinic hours’ flexibility is important, our findings emphasize the importance of identifying subsets of children who should be prioritized for vaccination at biologically optimal times of the day.

The strength of our study relies on high-level national data capturing all children vaccinated against varicella with long-term follow-up that captures future breakthrough infections. Our analysis has limitations. First, as with any observational study, patients were not randomly assigned to specific vaccination times, and demographic differences between groups can bias results. Second, we were not able to reach conclusions regarding disease severity because of the limited number of hospitalizations and the difficulty of distinguishing between varicella-related hospitalizations or hospitalizations for other causes. Third, our dataset lacks viral PCR testing and solely depends on coding diagnosis; however, varicella diagnosis is mainly clinical, especially in the outpatient setting. Fourth, our results reflect the cumulative behavior of our cohort and were not able to detect interindividual differences.

In summary, our study provides compelling evidence of a substantial association between the timing of varicella vaccination and its clinical efficacy in preventing breakthrough infections. These findings underscore the importance of considering circadian rhythms in vaccination strategies and highlight the potential for optimizing vaccine effectiveness by administering vaccines at biologically optimal times of day. Importantly, our study builds upon previous research, demonstrating similar findings regarding the timing of COVID-19 vaccination and its efficacy, further strengthening the relevance and generalizability of our results.

## Methods

### Sex as a biological variable.

Our study examined male and female children, and similar findings are reported for both sexes.

### Study design and setting.

This retrospective cohort study was conducted using the Clalit Health Maintenance Organization (HMO) Data Sharing Platform, powered by MDClone (https://www.mdclone.com). This platform utilizes advanced algorithms to deidentify and extract data from electronic medical records, ensuring both data quality and patient privacy. Clalit, the largest HMO in Israel, provides medical services to approximately 5.4 million individuals, representing 52% of the country’s population. The Clalit HMO database captures all clinical encounters, diagnoses, medications, and laboratory data for its participants anywhere within the country, regardless of setting, including all clinical activity and diagnostic testing. Since this was a retrospective study, patient informed consent was not required.

### Study period.

We analyzed data from January 2002 to December 2023. The varicella vaccine was introduced to the private market in Israel in 2003 with vaccine coverage of less than 30% and to the National Immunization Program (NIP) in September 2008. In the NIP, the varicella vaccine was administered as 2 doses, first at the age of 1 year and then at 6–7 years, to children born after January 1, 2007. To children born between January 1, 2002, and December 31, 2006, 2 doses were given with a 6-week interval at the age of 6–7 years. Vaccine coverage rapidly reached over 90% after its introduction to the NIP (25). The second dose of the varicella vaccine is given as part of a school vaccination campaign, exclusively during the morning.

Therefore, we extracted data from all Clalit HMO members younger than 6 years who received the first dose of varicella vaccine and joined Clalit HMO prior to January 2002, ensuring a complete medical history was on file. The timestamp for vaccine administration was determined by the time of the nurse visit encounter. Children with documented vaccination visit times between 20:00 and 7:00 (1,015, 0.4%) were excluded to avoid inadequate data. Furthermore, 18 children were excluded because of missing data on their sex (Figure 1).

### Data sources and organization.

We analyzed deidentified patient-level data extracted from Clalit HMO electronic records. Continuous variables encompassed age at immunization, time and date of vaccine administration, and weight. Dichotomous variables comprised sex (male vs. female), ethnicity (Jewish vs. non-Jewish), and comorbidities associated with varicella infection risk (26–29) and vaccine effectiveness (30, 31), defined by specific codes indicating primary immune deficiency, malignancy, transplantation, and chemotherapy administration grouped as immunodeficiency. Obesity, a known independent factor associated with nonresponse to varicella vaccination (32), was determined either by coding or by weight exceeding the 95th percentile for age and sex. All specific codes are detailed in Supplemental Table 1.

### Study outcome.

The primary outcome was varicella infection as defined by International Classification of Diseases 10 codes for the above surrogate for varicella breakthrough infection. Infections in the first 14 days following vaccination were excluded from the analysis, since patients during this interval are not considered to have a complete immunologic response to vaccination.

### Statistics.

An initial descriptive analysis included calculations of single-variable distribution, central tendency, and dispersion. We stratified vaccine timing in 4-hour bins: morning hours (7:00–10:59), late morning to afternoon (11:00–15:59), or evening hours (16:00–19:59) for comparison groups (Figure 2).

Kaplan-Meier analysis with a log-rank test was used for the univariate analysis. A Cox proportional hazards regression model was used to estimate the association between vaccine administration time of day and study endpoint. We treated the first varicella infection or the administration of a second dose of varicella vaccine as a terminal event, whichever came first. Recurrent infections were not considered in our analysis.

We conducted sensitivity analysis using 8:00–9:59 as a reference for the Cox regression model, comparing this reference time bin with successive 2-hour intervals across the day, incrementing by 1 hour with each iteration. Our hypothesis was that a diurnal rhythm in vaccine efficacy should produce a sinusoidal trend in HRs as a function of vaccination time.

We performed all statistical analyses using R version 3.5.0. Code for all analyses is available in Supplemental Data 1. A *P* value of less than 0.05 was used to indicate significance in all analyses.

### Study approval.

The Soroka University Medical Center and Clalit Data Extraction Ethical Committee (Beer Sheva, Israel) approved the study with number 0367-22.

### Data availability.

Deidentified data will be made available upon reasonable request to the corresponding author. Code for R (Supplemental Data 1) and Supporting Data Values are included in the supplement.

## Author contributions

DD conceptualized and designed the study and drafted the initial manuscript. YK coordinated data collection and data analysis. JAH conceptualized the study and critically reviewed the manuscript. GH conceptualized and designed the study and critically reviewed the manuscript. All authors approved the final manuscript.

## Supplementary Material

Supplemental data

ICMJE disclosure forms

Supporting data values

## Figures and Tables

**Table 1:**
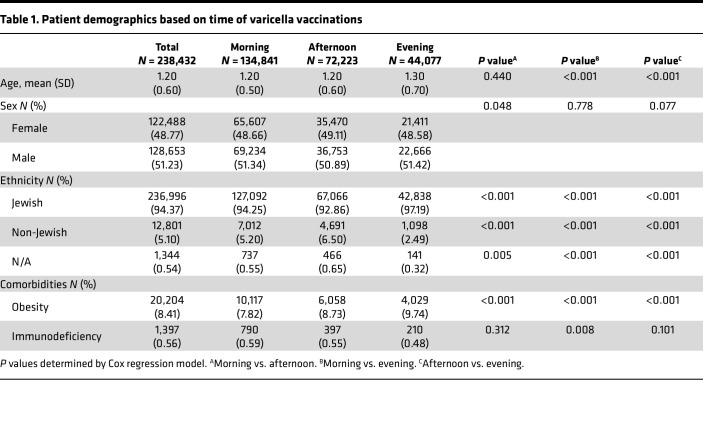
Patient demographics based on time of varicella vaccinations

**Table 2:**
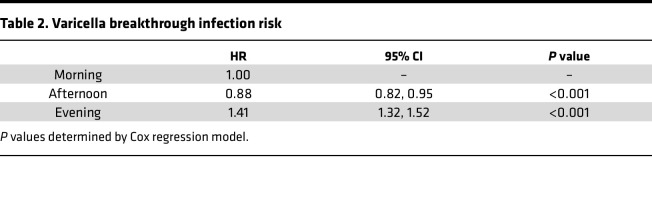
Varicella breakthrough infection risk

## Version 1 09/03/2024

In-Press Preview

## Version 2 10/22/2024

Electronic publication

## References

[1] Allada R, Bass J (2021). Circadian mechanisms in medicine. N Engl J Med.

[2] Cederroth CR (2019). Medicine in the fourth dimension. Cell Metab.

[3] Man K (2016). Immunity around the clock. Science.

[4] Wang C (2022). The circadian immune system. Sci Immunol.

[5] Holtkamp SJ (2021). Circadian clocks guide dendritic cells into skin lymphatics. Nat Immunol.

[6] Cervantes-Silva MP (2022). The circadian clock influences T cell responses to vaccination by regulating dendritic cell antigen processing. Nat Commun.

[7] Fortier EE (2011). Circadian variation of the response of T cells to antigen. J Immunol.

[8] Nobis CC (2019). The circadian clock of CD8 T cells modulates their early response to vaccination and the rhythmicity of related signaling pathways. Proc Natl Acad Sci U S A.

[9] Langlois PH (1995). Diurnal variation in responses to influenza vaccine. Chronobiol Int.

[10] Zhang H (2021). Time of day influences immune response to an inactivated vaccine against SARS-CoV-2. Cell Res.

[11] Hazan G (2023). Biological rhythms in COVID-19 vaccine effectiveness in an observational cohort study of 1.5 million patients. J Clin Invest.

[12] Huang WC (2011). Varicella breakthrough infection and vaccine effectiveness in Taiwan. Vaccine.

[13] Zhang X (2012). The epidemiology and risk factors for breakthrough varicella in Beijing Fengtai district. Vaccine.

[14] Mok H (2024). Circadian immunity from bench to bedside: a practical guide. J Clin Invest.

[15] De Bree LCJ (2020). Circadian rhythm influences induction of trained immunity by BCG vaccination. J Clin Invest.

[16] Karabay O (2008). Influence of circadian rhythm on the efficacy of the hepatitis B vaccination. Vaccine.

[17] Long JE (2016). Morning vaccination enhances antibody response over afternoon vaccination: a cluster-randomised trial. Vaccine.

[18] Phillips AC (2008). Preliminary evidence that morning vaccination is associated with an enhanced antibody response in men. Psychophysiology.

[19] Cowling BJ (2019). Influenza hemagglutination-inhibition antibody titer as a mediator of vaccine-induced protection for influenza B. Clin Infect Dis.

[20] Yang ZR (2023). Efficacy of SARS-CoV-2 vaccines and the dose-response relationship with three major antibodies: a systematic review and meta-analysis of randomised controlled trials. Lancet Microbe.

[21] Boivin DB (2022). Disturbance of the circadian system in shift work and its health impact. J Biol Rhythms.

[22] Hou Y (2020). Association between circadian disruption and diseases: a narrative review. Life Sci.

[23] Falconer M (2018). Improving vaccine coverage in adolescence and beyond. Hum Vaccin Immunother.

[24] Lafnitzegger A, Gaviria-Agudelo C (2022). Vaccine hesitancy in pediatrics. Adv Pediatr.

[25] Stein-Zamir C, Israeli A (2017). Age-appropriate versus up-to-date coverage of routine childhood vaccinations among young children in Israel. Hum Vaccin Immunother.

[26] Adler AL (2008). An outbreak of varicella with likely breakthrough disease in a population of pediatric cancer patients. Infect Control Hosp Epidemiol.

[27] Brown AEC (2016). Incidence and consequences of varicella in children treated for cancer in Guatemala. World J Pediatr.

[28] Danino D (2021). Hospitalizations for vaccine-preventable infections among pediatric hematopoietic cell transplantation recipients in the first 5 years after transplantation. Bone Marrow Transplant.

[29] Xiao P (2022). A prospective multicenter study on varicella-zoster virus infection in children with acute lymphoblastic leukemia. Front Cell Infect Microbiol.

[30] Chong PP, Avery RK (2017). A comprehensive review of immunization practices in solid organ transplant and hematopoietic stem cell transplant recipients. Clin Ther.

[31] Murata K (2020). Reduction in the number of varicella-zoster virus-specific T-cells in immunocompromised children with varicella. Tohoku J Exp Med.

[32] Chong CH (2023). Seroprevalence of varicella-zoster virus antibody and immunogenicity of live attenuated varicella vaccine in healthcare workers in Taiwan. J Microbiol Immunol Infect.

