# Supplementary material for: Diurnal rhythms in varicella vaccine effectiveness
Source: JCI Insight. 2024 Sep 3;9(20):e184452. doi: 10.1172/jci.insight.184452 (PMC11530121; doi:10.1172/jci.insight.184452)
Supplement: Supplemental data [file jciinsight-9-184452-s277.pdf]

Supplementary Figure 1

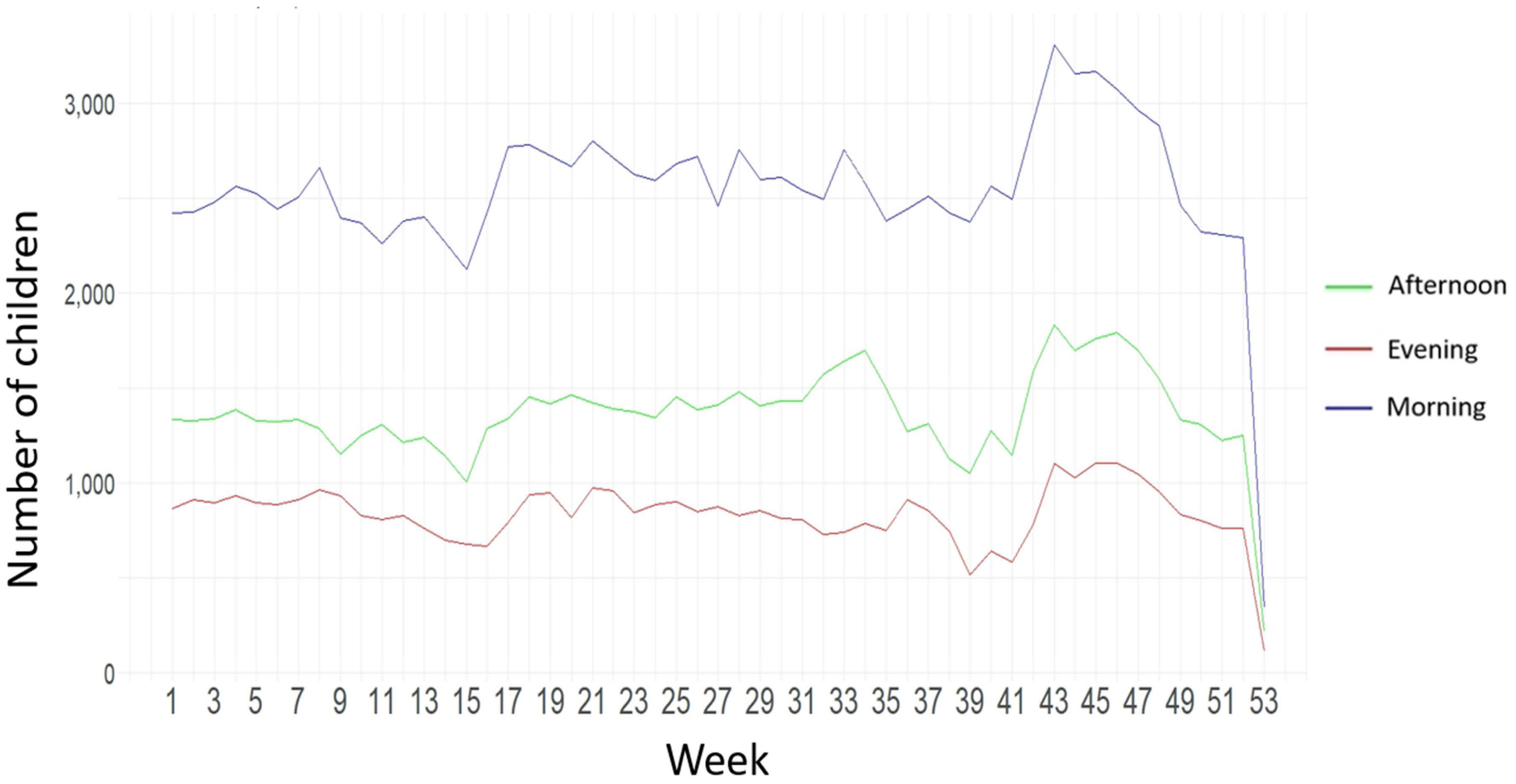

Supplementary Figure 1: Number of varicella infections by week of occurrence

Supplementary Table 1: ICD-10 Codes

Diagnosis	
Breakthrough infection	Varicella – B01<br>Chickenpox – B01<br>Varicella without mention any complications – B01.9
Obesity	Morbid obesity – E66.01<br>BMI> 30 – E66.9<br>Obesity –E 66.9
Vaccine	MMRV – 90710<br>Varicella – 90716
Immunodeficiency	Squamous cell carcinoma of skin of ear and externa auditory canal- C44.221<br>Squamous cell carcinoma of skin of lip – C44.02<br>Unspecified malignant neoplasm of skin of lip – c44.00<br>Unspecified organ or tissue replaced by transplant – z94.9<br>Splenectomy – z90.81<br>Wiskott – Aldrich syndrome – D82.0<br>Secondary malignant neoplasm of skin – C79.2<br>Secondary malignant neoplasm of lung – C78.0<br>Secondary malignant neoplasm of kidney – c79.0<br>Secondary malignant neoplasm of lymph node – head\neck – c77.0 , c79.89<br>Secondary malignant neoplasm of lymph – c77<br>stomach cancer – c16.9<br>non-Hodgkin lymphoma / mycosis fungoides – c84.00<br>myelodysplastic syndrome – D46.9<br>myelo lymphoproliferative syndrome – D47.9<br>multiple myeloma – c90.00<br>melanoma – c43.9<br>malignancy of uterus –c55<br>malignancy of unknown site – c80.1<br>malignancy of thyroid – c73<br>malignancy of pharynx – c14.00<br>malignancy of pancreas – c25.9<br>malignancy of other site – c80.1<br>malignancy of other male/ female genital organs – c80.1 , c49.59<br>malignancy of lung – c34.90<br>malignancy of liver / bile ducts – c22.1<br>malignancy of larynx – c32<br>malignancy of kidney – c64.9<br>malignancy of esophagus – c15.9<br>malignancy of connective tissue/ sarcoma – c49.9<br>basal cell carcinoma of skin of lip – c44.01<br>basal cell carcinoma of skin of ear and external auditory canal – c44.221<br>aleukemic leukemia myeloid – c92.z0<br>aleukemic leukemia lymphoid – c91.z0<br>2 nd and unsp mal neoplasm of lymph node ing.region+ lower limb –c77.4, c79.89<br>2nd and unsp. mal neoplasm intrapelvic lymph nodes – c77.5<br>Bone marrow replaced by transplant – z94.81<br>Burkitt's tumor or lymphoma – c83.7<br>Bone marrow transplant – z94.81<br>Carcinoma in situ – vagina\ vulva – D07.01 D07.02<br>Chemotherapy treatment – non specified.<br>Chronic lymphocytic leukemia – c91.90<br>Combined immunity deficiency – D81<br>Common variable immunodeficiency – D83<br>Complement deficiency disorders – D84.1<br>Disseminated malignant neoplasm – c80.0<br>Heart replaced by transplant – z94.1

Hodgkins's disease- c81
Heart transplant – z94.1
Immune deficiency disorders – D84.9
Jobs syndrome – D82.4
Kaposi sarcoma of soft tissue 1992 – c46.1
Kidney replaced by transplant - z94.0
Leukemia – c95.9
Leukemia of unspecified cell type – c95
Leukemia reticuloendotheliosis –c91.4
Liver replaced by transplant –z94.4
Lung replaced by transplant- z94.2
Lymphoid leukemia subacute –c91.z
Lymphoid leukemia acute in remission 1992 – c91.91
Lymphoma nodular – c85.80
Lymphoma unspecified – c85.90
lymphosarcoma – c83.50
lymphosarcoma unspecified site –c85.90
liver transplant – z94.4
lung transplant – z94.2
mal neo eyeball except conjunctiva cornea retina choroid –c69.90
malignant neoplasm of ill-defined sites within the respiratory system –c39.9
malignant neoplasm of other specified sites of female genital organs –c80.1
malignant neoplasm of skin of ear and external auditory canal – c44.201
malignant neoplasm of convective and soft tissue site unspecified- c49.9
malignant neoplasm of other specified sites of female genital organs-c80.1 c49.59
malignant neoplasm colon rectum – c18.9
malignant neoplasm pancreas – c25.9
malignant neoplasm stomach – c16.9
malignant neoplasm – c80.1
malignant neoplasm anal canal – c21.1
malignant neoplasm associated with transplanted organ – c80.2
malignant neoplasm bile duct ampulla vater – c24.0, c25.3
malignant neoplasm bladder-c67
malignant neoplasm bronchus and trachea –c34.90
malignant neoplasm bronchus/lung – c34.90
malignant neoplasm cervix – c53.9
malignant neoplasm choroid –c69.3
malignant neoplasm colon appendix – c18.9
malignant neoplasm colon ascending colon –c18.2
malignant neoplasm colon descending colon –c18.6
malignant neoplasm colon transverse colon-c18.4
malignant neoplasm ear cartilage – c49.0
malignant neoplasm ectopic undescended testis –c62.00
malignant neoplasm endometrium –c54.1
malignant neoplasm esophagus unspecified – c15.9
malignant neoplasm fallopian tube – c57.0
malignant neoplasm of frontal sinus – c31.2
malignant neoplasm hypopharynx –c13
malignant neoplasm lacrimal gland –c69.5
malignant neoplasm larynx –c32
malignant neoplasm liver primary – c22.0
malignant neoplasm lung unspecified – c34.90
malignant neoplasm mandible –c41.1
malignant neoplasm maxillary sinus-c31.0
malignant neoplasm middle ear – c30.1
malignant neoplasm nasopharynx – c11
malignant neoplasm of abdominal esophagus –c15.5
malignant neoplasm of adrenal gland –c74
malignant neoplasm of anterior portion of floor of mouth – c04.0
malignant neoplasm of anus, unspecified – c21.0
malignant neoplasm of aortic body and other paraganglia – c75.5
malignant neoplasm of body of pancreas –c25.1
malignant neoplasm of body of stomach – c16.2

malignant neoplasm of brain - c71
malignant neoplasm of brain , unspecified – c71.9
malignant neoplasm of brain stem – c71.7
malignant neoplasm of cardia – c16.0
malignant neoplasm of cecum –c18.0
malignant neoplasm of cervical esophagus – c15.3
malignant neoplasm of cervix uteri, unspecified –c53.9
malignant neoplasm of conjunctiva – c69.0
malignant neoplasm of duodenum –c17.0
malignant neoplasm of extrahepatic bile ducts –c24.0
malignant neoplasm of female genital organ , site unspecified –c57.9
malignant neoplasm of floor of mouth – c04
malignant neoplasm of fundus of stomach – c16.1
malignant neoplasm of gallbladder – c23
malignant neoplasm of glottis – c32
malignant neoplasm of head of pancreas – c25.0
malignant neoplasm of hepatic flexure –c18.3
malignant neoplasm of ileum – c17.2
malignant neoplasm of intrahepatic bile ducts –c22.1
malignant neoplasm of jejunum – c17.1
malignant neoplasm of kidney except pelvis – c64.9
malignant neoplasm of labia majora – c51.0
malignant neoplasm of laryngeal cartilages –c32.3
malignant neoplasm of larynx unspecified – c32.9
malignant neoplasm of lateral portion of floor of mouth – c04.1
malignant neoplasm of lower gum – c03.1
malignant neoplasm of lower lobe bronchus or lung – c34.3
malignant neoplasm of lower third of esophagus – c15.5
malignant neoplasm of main bronchus – c34.0
malignant neoplasm of middle third of esophagus –c15.4
malignant neoplasm of nasal cavities – c30.0
malignant neoplasm of nipple and areola of male breast –c50.029
malignant neoplasm of orbit – c69.6
malignant neoplasm of oropharynx – c10.0
malignant neoplasm of other and unspecified testis – c62.10
malignant neoplasm of other major salivary glands – c08.9
malignant neoplasm of other parts of bronchus or lung –c34.80
malignant neoplasm of other sites of floor of mouth –c04.8
malignant neoplasm of other specified parts of mouth – c06.89
malignant neoplasm of other specified sites of larynx – c32.8
malignant neoplasm of other specified sites of female breast – c50.819
malignant neoplasm of other specified sites of small intestine –c17.8
malignant neoplasm of ovary – c56
malignant neoplasm of pancreas –c25
malignant neoplasm of pancreatic duct – c25.3
malignant neoplasm of parathyroid gland –c75.0
malignant neoplasm of parotid gland –c07
malignant neoplasm of penis and other male organ –c60.8
malignant neoplasm of peritoneum unspecified –c48.2
malignant neoplasm of pituitary gland and craniopharyngeal duct –c75.1
malignant neoplasm of prostate – c61
malignant neoplasm of pyloric antrum – c16.3
malignant neoplasm of pylorus – c16.4
malignant neoplasm of rectosigmoid junction –c19
malignant neoplasm of rectum – c20
malignant neoplasm of retina –c69.2
malignant neoplasm of retromolar area –c06.2
malignant neoplasm of scrotum – c63.2
malignant neoplasm of sigmoid colon –c18.7
malignant neoplasm of small intestine unspecified – c17.9
malignant neoplasm of splenic flexure –c18.5
malignant neoplasm of stomach – c16
malignant neoplasm of submandibular gland –c08.0

malignant neoplasm of sublingual gland –c08.1
malignant neoplasm of tail of pancreas – c25.2
malignant neoplasm of thoracic esophagus –c15.4
malignant neoplasm of tongue – c02.9
malignant neoplasm of trachea – c33
malignant neoplasm of upper gum –c03.0
malignant neoplasm of upper lobe bronchus or lung –c34.1
malignant neoplasm of upper third of esophagus –c15.3
malignant neoplasm of uterus part unspecified – c55
malignant neoplasm of vallecula epiglottic –c10
malignant neoplasm of vestibule of mouth –c06.1
malignant neoplasm of vulva unspecified –c51.9
malignant neoplasm of pancreas islet Langerhans –c25.9
malignant neoplasm pancreas unspecified – c25.9
malignant neoplasm pleura – c38.4
malignant neoplasm secondary bone marrow – c79.51
malignant neoplasm secondary brain spinal cord –c79.31,c79.51
malignant neoplasm secondary liver –c78.7
malignant neoplasm secondary peritoneum –c78.6
malignant neoplasm sphenoidal sinus – c31.3
malignant neoplasm supraglottic –c32.1
malignant neoplasm without specification of site –c80
medullary carcinoma of thyroid – c73
melanoma of choroid –c69.3
melanoma of labia minora – c51.9
melanoma of scrotum – c43.9
melanoma of skin site unspecified – c43.9
melanoma of vagina –c52
melanoma of vulva –c51.9
meningioma brain or spinal cord malignant –c70.9,c71.9,c72.0,d32.0.d32.1
metastatic carcinoma – c80.1
monocytic leukemia chronic –c93.1
monocytic leukemia acute without mention of remission 92 –c92.0,c93.90
multiple myeloma –c90
mycosis fungoides –c84.0
myelodysplastic syndrome with 5q deletion –d46.c
myeloid leukemia acute –c92.4
myeloid leukemia chronic - c92
myeloid leukemia subacute –c92.z
myeloid sarcoma without mention of remission 1992-c92.30
need for other prophylactic chemotherapy –z41.8
neoplasm malignant gum –c03.9
neurofibromatosis malignant – d49.2, Q85.00
osteosarcoma pagets disease - c41.9 ,M88.9
other malignant neoplasm urinary tract – c68.8
other malignant neoplasm of skin –c44.90
other malignant neoplasm of skin of lip – c44.0
other malignant neoplasm of skin site unspecified –c44.90
other malignant neoplasms –c80.1
other specified organ or tissue replaced by transplant –z94.9
other specified leukemia –c94
other specified malignant neoplasm of skin of ear and external auditory canal-c44.201
other specified malignant neoplasm of skin of lip – c44.09
other unspecified neoplasms –d49.9
other transplant – non specified
pancreas replaced by transplant – z94.83
poisoning by antineoplastic and immunosuppressive drugs –t45.1x4
polyp stomach malignant –c16.9 , k31.7
pancreas transplant – non specified.
reticulosarcoma – c83.3
acute leukemia –c95.00
breast cancer – c50.9
shronic leukemia –c95.10

	hodgkins lymphoma – c85.90<br>malignancy of bladder – c67.9<br>malignancy of bone –c41.9<br>malignancy of brain/cns – c71.9<br>malignancy of cervix uteri –c53.9<br>malignancy of colon or rectum-c20,c18.9<br>malignancy of connective tissue / sarcoma –c49.9

Supplementary Table 2

	Hazard Ratio	95% CI	*P* value
Obesity			
No	1·00	——	——
Yes	1·25	1·12, 1·39	<0·001
Immunodeficiency			
No	1·00	——	——
Yes	1·04	0·67, 1·61	0·9
Ethnicity			
Non-Jewish	1·00	——	——
Jewish	4·72	3·40, 6·56	<0·001
Sex			
Female	1·00	——	——
Male	1·16	1·09, 1·24	<0·001

CI: confidence interval

R - code

```
rm(list=is())
Sys.setlocale(category = "LC_ALL", locale = "Hebrew")
library(dplyr)
library(data.table)
library(readr)
library(tidyverse)
library(stringr)
library(lubridate)
library(readxl)
library(childsds)
library(gtsummary)
library(zoo)
library(flextable)
library(survival)
library(survminer)
library(ggplot2)
library(scales)
table = read.csv("//10.100.117.3/Projects/R02-Darom/R02-
Yoav_Kalron/tttable.csv")




table = table %>% filter(gender != "NULL") ## exclusion people
who without gender
table = table %>% filter(table$visitclinic.age < 6)  ## exclusion
people who get first vaccion after age 6
table = table %>%
filter(is.na(table$second_vaccine.vaccination.date.days.from.refe
rence) |

table$second_vaccine.vaccination.date.days.from.reference> 28) ##
exclusion people who have no logical second vaccion
table = table %>%
filter(table$sickness.start.date.days.from.reference  > 14
                          |
is.na(table$sickness.age.at.diagnosis))   ### exclusion people
who got sick 14 days after get the vaccion


table <- table[, !(names(table) == "sickness.diagnosis.type")]
table <- table[, !(names(table) ==
"second_vaccine.community.visits...age")]
table <- table[, !(names(table) ==
"econd_vaccine.community.visits...visit.date")]
table <- table[, !(names(table) ==
"second_vaccine.vaccines...patient.id")]
table <- table[, !(names(table) ==
"second_vaccine.vaccines...vaccination.date.days.from.reference")
]
table <- table[, !(names(table) ==
"second_vaccine.community.visits...visit.date")]
table <- table[, !(names(table) ==
"second_vaccine.vaccines...vaccine.name")]
table <- table[, !(names(table) == "immigration.date")]
```

```
table <- table[, !(names(table) == "mailing.language")]
table <- table[, !(names(table) ==
"second_vaccine.vaccines...vaccination.date")]
table <- table[, !(names(table) ==
"immunodeficiency.diagnosis.code")]
table <- table[, !(names(table) ==
"immunodeficiency.diagnosis.type")]
table <- table[, !(names(table) == "obisity.diagnosis.type")]

####
### group devision


group <- ifelse(table$visitclinic.visit.date.copy.hour >= 7 &
table$visitclinic.visit.date.copy.hour < 11 ,"morning",
                ifelse(table$visitclinic.visit.date.copy.hour >=
11 & table$visitclinic.visit.date.copy.hour < 16,"afternoon" ,

ifelse(table$visitclinic.visit.date.copy.hour >= 16&
table$visitclinic.visit.date.copy.hour < 20 ,"evening" ,"")))
table$group = group
table = table %>% filter(group != "")
table$group <- as.factor(table$group)


### obisity and immuonodeficiency yes \ no

table$immunodeficiency.diagnosis =
  ifelse(is.na(table$immunodeficiency.age.at.diagnosis), "no",
"yes")

c = sum(table$immunodeficiency.diagnosis == "yes" , na.rm = T)
c

table$obisity.diagnosis =
ifelse(is.na(table$obisity.age.at.diagnosis) &
                                   table$wight.bmi.percentage <
95, "no" , "yes")

b = sum(table$obisity.diagnosis == "yes", na.rm = t )
b
### histogram hours


format_with_commas <- function(x) {
  return(format(x, big.mark = ","))
}
ggplot(data = table, aes(x = visitclinic.visit.date.copy.hour)) +
  geom_histogram(bins = 25, color = "black") +
  labs(x = "Hour", y = "Frequency", title = "Histogram of Hours")
+
  scale_x_continuous(breaks = seq(0, 23, by = 1)) +  # Customize
x-axis ticks
  scale_y_continuous(breaks = c(0, 10000, 20000, 30000, 40000,
50000),
                     labels = format_with_commas)
```

```
d = sum(!is.na(table$sickness.age.at.diagnosis) &
!is.na(table$second_vaccine.age.at.event))
d


###seasons graph
extract_day_month_year <- function(datetime_string) {
  substr(datetime_string, 1, 10)
}


table = table %>%
  mutate(only_date = sapply(visitclinic.visit.date,
extract_day_month_year))

table <- table %>%
  mutate(only_date = as.Date(only_date, format = "%d/%m/%Y"))

table <- table %>%
  mutate(week = week(only_date))

count_data <- table %>%
  group_by(week, group) %>%
  summarise(count = n())


p= ggplot(count_data, aes(x = week, y = count, color = group)) +
  geom_line() +
  labs(x = "Week", y = "Count", title = "Occurrences of Weeks by
Group") +
  scale_x_continuous(breaks = seq(min(count_data$week),
max(count_data$week), by = 2)) + scale_y_continuous(labels =
comma) +
  scale_color_manual(values = c("morning" = "blue", "afternoon" =
"green", "evening" = "red")) +
  theme_minimal()
p <- p + theme(axis.text.x = element_text(size = 30))
p <- p + theme(axis.text.y = element_text(size = 24))
p <- p + theme(plot.margin = margin(0, 0, 0, 0))
p <- p + theme(axis.text.x = element_text(margin = margin(b =
10)))
p <- p + theme(axis.text.y = element_text(margin = margin(l =
10)))

p






##change sector
table$marketing.sector <- gsub("חרדים", "jewish",
table$marketing.sector)
table$marketing.sector <- gsub("עולים", "na",
table$marketing.sector)
```

```
table$marketing.sector <- gsub("כללי", "jewish",
table$marketing.sector)
table$marketing.sector <- gsub("דתי מעורב", "jewish",
table$marketing.sector)
table$marketing.sector <- gsub("ערבים", "arabic",
table$marketing.sector)
table$marketing.sector <- gsub("לא ידוע", "na",
table$marketing.sector)
table$marketing.sector <- gsub("אנגלית", "na",
table$marketing.sector)



###table1

table <- as.data.frame(table)

summary_table =  table %>% tbl_summary(by = group ,include =
c(visitclinic.age
,gender,marketing.sector,obisity.diagnosis,immunodeficiency.diagn
osis),
                                       statistic =
list(all_continuous()~ c("{mean} ± {sd}",

"{median} ({p25}, {p75})"),

all_categorical()~ "{n} ({p}%)"),
                                       missing = "no", digits =
list(all_categorical() ~ c(0,2),

all_continuous() ~ c(1,1,0)),
                                       type = all_continuous() ~
'continuous2')  %>%


  modify_caption("Table 1") %>%
  bold_labels()  %>%
  add_overall() %>%
  add_p(all_continuous() ~ "aov")  ### did manually
summary_table

# Define the observed frequencies
group_a_total <- 134841
group_a_sick <- 69234
group_b_total <- 44077
group_b_sick <- 22666

contingency_table <- matrix(c(group_a_sick, group_b_sick,
                              group_a_total - group_a_sick,
group_b_total - group_b_sick),
                            nrow = 2,
                            dimnames = list(c("Sick", "Not
Sick"), c("Group A", "Group B")))

# Perform chi-square test
chi_square_result <- chisq.test(contingency_table)
p_value <- chi_square_result$p.value
print(p_value)
```

```
### for age

# Assuming you have the means and sample sizes of both groups
mean_group_a <- 1.2  # Replace with the mean of Group A
mean_group_b <- 1.3  # Replace with the mean of Group B
sample_size_group_a <- 72223
sample_size_group_b <-44077

# Perform t-test
t_test_result <- t.test(x = rnorm(sample_size_group_a,
mean_group_a),
                        y = rnorm(sample_size_group_b,
mean_group_b),
                        alternative = "two.sided",
                        var.equal = FALSE,
                        paired = FALSE)

p_value <- t_test_result$p.value
print(p_value)

###for meadian
morning_ages <- table[table$group == "morning",
"visitclinic.age"]
morning_ages = as.vector(morning_ages)
afternoon_ages <- table[table$group == "afternoon",
"visitclinic.age"]
afternoon_ages = as.vector(afternoon_ages)
evening_ages <- table[table$group == "evening",
"visitclinic.age"]
evening_ages = as.vector(evening_ages)
result <- wilcox.test(evening_ages,afternoon_ages)
result

##### cox

table$event =ifelse(!is.na(table$sickness.age.at.diagnosis)
                    & table$sickness.age.at.diagnosis < 6
                    ,1,0)
### kids who get sick befor the age 6


table$time_to_event=
  ifelse(table$event == 1 ,
table$sickness.start.date.days.from.reference,
         (6 -
table$cohort.reference.event.vaccines...age.at.event) *365
  )


###    (6 - table$cohort.reference.event.vaccines...age.at.event)
*365
### the number of days for kids who dont got sick until age 6
from the vaccitation day

table$time_to_event =
  ifelse(!is.na(table$death.deceased.date.days.from.reference) &
table$event == 0,
```

```
table$death.deceased.date.days.from.reference,table$time_to_event
)

### kids who got dead during the study and dont got sick before.



table$event = ifelse(
!is.na(table$second_vaccine.vaccination.date.days.from.reference)
& table$second_vaccine.vaccination.date.days.from.reference <
table$time_to_event
                     ,0,table$event)

### if the kids get second vaccion before sickness day - event =
0

table$time_to_event =
  ifelse(
!is.na(table$second_vaccine.vaccination.date.days.from.reference)
& table$second_vaccine.vaccination.date.days.from.reference <
table$time_to_event

,table$second_vaccine.vaccination.date.days.from.reference,table$
time_to_event)

### if kids got second vaccion  brfore the time they got sick ,
### the time to event will be until the second vaccion


###cox regression univariate

table$group <- relevel(table$group, ref = "morning")

cox_model <- coxph(Surv(time_to_event
                        , event)
                   ~ group     , data = table)

tbl_regression(cox_model,exponentiate =
T,add_estimate_to_reference_rows = T)

###cox regression multivariate

table$group <- relevel(table$group, ref = "morning")

cox_model <- coxph(Surv(time_to_event
                        , event)
                   ~ group + obisity.diagnosis +
immunodeficiency.diagnosis + marketing.sector + gender    , data
= table)

tbl_regression(cox_model,exponentiate =
T,add_estimate_to_reference_rows = T)




##### graph by group
```

```
my_table = as.data.frame(table)

surv_model = survfit(Surv(time_to_event, event) ~ group    , data
= my_table)

graph=  ggsurvplot(fit = surv_model, conf.int = F, palette =
"jama",
                   risk.table = F , risk.table.height = 0.3,
cumevents = F, cumevents.height = 0.3
                   , surv.scale = "percent",
                   xlab = "Time since vaacine
(days)",ylab="Cumulative incidence (%)",
                   ylim = c(0.97,1) , title = "", font.x = c(30,
"plain", "black"),font.y = c(30, "plain", "black"),
                   censor = T, legend = "right",legend.title =
"",font.tickslab = c(27, "plain", "black"))
graph


##### graph by group + obisity


my_table = as.data.frame(table)

surv_model = survfit(Surv(time_to_event, event) ~ group +
obisity.diagnosis   , data = my_table)

graph=  ggsurvplot(fit = surv_model, conf.int = F, palette =
"jama",
                   risk.table = F , risk.table.height = 0.3,
cumevents = F, cumevents.height = 0.3
                   , surv.scale = "percent",
                   xlab = "Time since vaacine
(days)",ylab="Cumulative incidence (%)",
                   ylim = c(0.97,1) , title = "", font.x = c(30,
"plain", "black"),font.y = c(30, "plain", "black"),
                   censor = T, legend = "right",legend.title =
"",font.tickslab = c(27, "plain", "black"))
graph


##### graph by group + sector
my_table = table %>% filter(marketing.sector != "na")



surv_model = survfit(Surv(time_to_event, event) ~ group +
marketing.sector   , data = my_table)

graph=  ggsurvplot(fit = surv_model, conf.int = F, palette =
"jama",
                   risk.table = F , risk.table.height = 0.3,
cumevents = F, cumevents.height = 0.3
                   , surv.scale = "percent",
                   xlab = "Time since vaacine
(days)",ylab="Cumulative incidence (%)",
```

```
                    ylim = c(0.97,1) , title = "", font.x = c(30, 
"plain", "black"),font.y = c(30, "plain", "black"),
                    censor = T, legend = "right",legend.title = 
"",font.tickslab = c(27, "plain", "black"))
graph

##### graph by group + sex


my_table = as.data.frame(table)

surv_model = survfit(Surv(time_to_event, event) ~ group + gender 
, data = my_table)

graph=  ggsurvplot(fit = surv_model, conf.int = F, palette = 
"jama",
                    risk.table = F , risk.table.height = 0.3, 
cumevents = F, cumevents.height = 0.3
                    , surv.scale = "percent",
                    xlab = "Time since vaacine 
(days)",ylab="Cumulative incidence (%)",
                    ylim = c(0.97,1) , title = "", font.x = c(30, 
"plain", "black"),font.y = c(30, "plain", "black"),
                    censor = T, legend = "right",legend.title = 
"",font.tickslab = c(27, "plain", "black"))
graph



##### graph by group + immunodefficencey


my_table = as.data.frame(table)

surv_model = survfit(Surv(time_to_event, event) ~ group + 
immunodeficiency.diagnosis   , data = my_table)

graph=  ggsurvplot(fit = surv_model, conf.int = F, palette = 
"jama",
                    risk.table = F , risk.table.height = 0.3, 
cumevents = F, cumevents.height = 0.3
                    , surv.scale = "percent",
                    xlab = "Time since vaacine 
(days)",ylab="Cumulative incidence (%)",
                    ylim = c(0.96,1) , title = "", font.x = c(30, 
"plain", "black"),font.y = c(30, "plain", "black"),
                    censor = T, legend = "right",legend.title = 
"",font.tickslab = c(27, "plain", "black"))
graph

######cox sinus

table$group2 <- ifelse(table$visitclinic.visit.date.copy.hour >= 
7 & table$visitclinic.visit.date.copy.hour < 9, "Group A",

ifelse(table$visitclinic.visit.date.copy.hour >= 9 & 
table$visitclinic.visit.date.copy.hour < 11, "Group C",
```

```
ifelse(table$visitclinic.visit.date.copy.hour >= 11 &
table$visitclinic.visit.date.copy.hour < 13, "Group E",

ifelse(table$visitclinic.visit.date.copy.hour >= 13 &
table$visitclinic.visit.date.copy.hour < 15, "Group G",

ifelse(table$visitclinic.visit.date.copy.hour >= 15 &
table$visitclinic.visit.date.copy.hour < 17, "Group I",


ifelse(table$visitclinic.visit.date.copy.hour >= 17 &
table$visitclinic.visit.date.copy.hour < 19, "Group K",

NA))))))


table$group3 <- ifelse(table$visitclinic.visit.date.copy.hour >=
7 & table$visitclinic.visit.date.copy.hour < 9, "Group A",

ifelse(table$visitclinic.visit.date.copy.hour >= 10 &
table$visitclinic.visit.date.copy.hour < 12, "Group D",

ifelse(table$visitclinic.visit.date.copy.hour >= 12 &
table$visitclinic.visit.date.copy.hour < 14, "Group F",

ifelse(table$visitclinic.visit.date.copy.hour >= 14 &
table$visitclinic.visit.date.copy.hour < 16, "Group H",

ifelse(table$visitclinic.visit.date.copy.hour >= 16 &
table$visitclinic.visit.date.copy.hour < 18, "Group J",

ifelse(table$visitclinic.visit.date.copy.hour >= 18 &
table$visitclinic.visit.date.copy.hour < 20, "Group L",

NA))))))



cox_model2 <- coxph(Surv(time_to_event
                         , event)
                    ~ group2 , data = table)

tbl_regression(cox_model2,exponentiate =
T,add_estimate_to_reference_rows = T)





totalhr = c (1,0,1.07,1.04,0.97,0.88,0.82
             ,0.86,1.15,1.42,
             1.55,1.70)
min_values =
c(0,0,0.98,0.95,0.88,0.79,0.72,0.75,1.04,1.3,1.41,1.46)
max_values=
c(0,0,1.16,1.13,1.07,0.99,0.94,0.97,1.27,1.55,1.72,1.97)
```

```
hours <- paste(formatC(7:18, width = 2, flag = "0"), " - ",
               formatC((7:18) + 2, width = 2, flag = "0"), sep =
"")
hours
dot_color <- "black"
line_color <- "blue"
background_color <- "lightgray"
title_color <- "black"
text_color <- "darkgray"
p= plot( totalhr, xlab = "", ylab = "", main = "Infection By
Hours", xaxt = "n",
         col = dot_color, pch = 16, cex =1.6, ylim = c(0.5,2.2),
bg = background_color,lwd = 2)
axis(1, at = seq_along(hours), labels = hours, las = 2,cex.axis =
1.45
)
abline(h = 1, lty = 2)
non_zero_lengths <- which(max_values != min_values)
arrows(x0 = non_zero_lengths, y0 = min_values[non_zero_lengths],
       x1 = non_zero_lengths, y1 = max_values[non_zero_lengths],
       angle = 90, code = 3, length = 0.05, col = "black")
```
